# Firing Dynamics and Modulatory Actions of Supraspinal Dopaminergic Neurons during Zebrafish Locomotor Behavior

**DOI:** 10.1016/j.cub.2014.12.033

**Published:** 2015-02-16

**Authors:** Michael Jay, Francesca De Faveri, Jonathan Robert McDearmid

**Affiliations:** 1 Department of Biology, College of Medicine, Biological Sciences and Psychology, University of Leicester, Leicester LE1 7RH, UK

## Abstract

**Background:**

Dopamine (DA) has long been known to have modulatory effects on vertebrate motor circuits. However, the types of information encoded by supraspinal DAergic neurons and their relationship to motor behavior remain unknown.

**Results:**

By conducting electrophysiological recordings from awake, paralyzed zebrafish larvae that can produce behaviorally relevant activity patterns, we show that supraspinal DAergic neurons generate two forms of output: tonic spiking and phasic bursting. Using paired supraspinal DA neuron and motoneuron recordings, we further show that these firing modes are associated with specific behavioral states. Tonic spiking is prevalent during periods of inactivity while bursting strongly correlates with locomotor output. Targeted laser ablation of supraspinal DA neurons reduces motor episode frequency without affecting basic parameters of motor output, strongly suggesting that these cells regulate spinal network excitability.

**Conclusions:**

Our findings reveal how vertebrate motor circuit flexibility is temporally controlled by supraspinal DAergic pathways and provide important insights into the functional significance of this evolutionarily conserved cell population.

## Highlights

• Supraspinal DAergic neurons generate tonic spiking and phasic bursting
• Tonic spiking correlates with periods of locomotor inactivity
• Phasic bursting correlates with periods of locomotor activity
• Targeted ablation of supraspinal DAergic neurons depresses locomotor output

## Teaser

Dopamine (DA) is a known modulator of motor circuits. Here, Jay et al. use the zebrafish to study in vivo activity patterns and functional roles of identified DAergic diencephalospinal neurons. Their findings provide important insights into the behavioral relevance of this evolutionarily conserved cell population.

## Introduction

Dopaminergic diencephalospinal neurons (DDNs) are an evolutionarily conserved population of forebrain neurons that provide the primary source of dopaminergic (DAergic) innervation to the vertebrate spinal cord. Numerous pharmacological and lesion studies have afforded insights into the possible function of DDNs. These suggest that dopamine (DA) released by these cells influences somatosensory processing [1, 2], autonomic output [3], locomotion [4–12], behavioral maturation [8, 13], and spinal network development [14]. However, one outstanding issue is that relationships between DDN activity and behavior have not been established. Thus, the behavioral contexts associated with spinal DA release remain unknown.

Zebrafish larvae are an ideal model for studying neurofunctional aspects of DDN physiology. These fish develop rapidly, and all DAergic tracts are established within the first 4 days of life [15], a stage when animals remain accessible to in vivo imaging and electrophysiology approaches. In zebrafish, the DDNs comprise large-diameter neurons in DC2/DC4 of the posterior tuberculum and medium-sized, cerebrospinal fluid-contacting cells within DC5 of the hypothalamus [15–17]. Conserved cell morphology, anatomical location, axonal projection patterns, and expression of the *orthopedia* homeobox gene [15, 18] strongly suggest that these neurons are homologous to the A11 DAergic cell cluster of the mammalian forebrain.

Here, we have used in vivo patch clamping to study endogenous activity patterns of DDNs recorded from DC2 of awake, paralyzed zebrafish larvae at 4 days post fertilization (dpf). We have examined the behavioral contexts that correlate with DDN activity and studied the behavioral effects of ablating these neurons. In doing so, we shed light on the role supraspinal DA neurons play in control of vertebrate behavior.

## Results

### Identification of DDNs for Patch Clamp Recording

**Figure 1:**
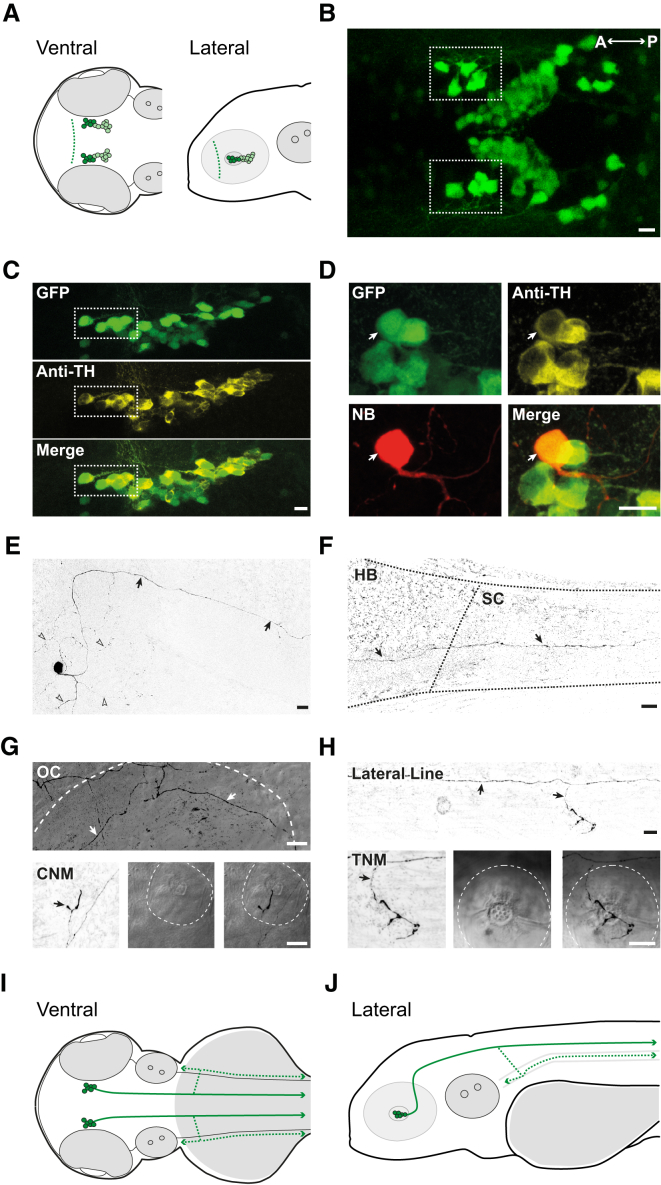
Identification of DDNs in the Diencephalon of *ETvmat2:GFP* Zebrafish Larvae at 4 dpf (A) Ventral (left) and lateral (right) schematic overview of DC2 (dark green) and DC4/5 (light green) neurons in the diencephalon (dashed line represents the anterior diencephalic border) of 4 dpf larvae. (B) Ventral view of GFP-labeled cells in the diencephalon. Prospective DDNs in DC2 comprise a small cluster of strongly fluorescent, large-diameter cells (dashed boxes) near the anterior aspect of the diencephalon. (C) Lateral images of the posterior tuberculum labeled with GFP (green, top) and anti-tyrosine hydroxylase antibodies (Anti-TH, yellow, middle). Merged image (bottom) reveals that all large, GFP-positive neurons in the anterior aspect of posterior tuberculum are immunoreactive for TH. (D) Lateral view of a GFP-expressing neuron in the anterior posterior tuberculum (green) labeled with neurobiotin (NB, red) and co-stained with anti-tyrosine hydroxylase antibodies (Anti-TH, yellow). Merged image (bottom right) reveals NB-labeled cells are GFP and TH positive. (E and F) Lower-magnification contrast inverted composite images of the same NB-labeled neuron in (D) depicting the soma and primary axon in the diencephalon (E; arrows) and the primary axon (F; arrows) extending from the hindbrain (HB) to the spinal cord (SC). Extensive axonal arborization is observed near the soma (arrow heads in E). (G and H) At the level of the hindbrain, the primary axon branches into the periphery, innervating the otic capsule (OC, top in G), cranial neuromasts (CNM, bottom in G), lateral line (top in H), and trunk neuromasts (TNM, bottom in H). White dashed lines in (G) and (H) mark the OC (top) and CNM and TNM (bottom). Bottom panels in (G) and (H) depict contrast-inverted image of NB labeling (left), bright field (middle), and merged NB-bright field images (right). (I and J) Ventral (I) and lateral (J) schematic overviews of DDNs and their arborization patterns. Axons project caudally into the spinal cord (solid green lines) and into the periphery (dashed green lines). In (B)–(H), anterior is left and posterior is right. In (C)–(H), dorsal is up and ventral is down. Scale bars in (B)–(H) represent 10 μm. (A), (I), and (J) are not to scale.

To identify DAergic neurons for study, we used *ETvmat2:GFP* zebrafish that express GFP in monoaminergic cell populations of the brain. Previous studies have shown that DAergic neurons are clearly observable in the diencephalon of *ETvmat2:GFP* larvae [19]. Further examination of GFP-labeled neurons in this region revealed a small cluster (5–7 neurons) of candidate DDNs in DC2, located toward the anterior border of the posterior tuberculum (Figures 1A and 1B). These cells could be readily distinguished from neighboring GFP-labeled neurons because they had large-diameter somas (10.19 ± 0.22 μm; n = 46 cells), were located in a stereotypical position, and were intensely fluorescent [15, 17, 20].

To confirm that the aforementioned cells were DAergic, we processed *ETvmat2:GFP* larvae for anti-tyrosine hydroxylase (TH) immunohistochemistry. All large, intensely fluorescent neurons in DC2 co-expressed TH (n = 6; Figure 1C). Although noradrenergic neurons also express TH, previous studies show that noradrenergic neurons are restricted to brainstem regions [15, 20]. Thus, as previously reported [15–17, 20], TH-positive cells in DC2 are DAergic neurons.

Juxtacellular neurobiotin labeling was used to label individual DC2 neurons so that axonal projection patterns could be studied. All labeled neurons (n = 3) branched extensively near the soma and extended a primary axon that first coursed dorsally before turning to project caudally (Figures 1D–1F). In agreement with previous studies [15], branching was also observed at the level of the hindbrain. Closer inspection revealed that these branches innervated the otic capsule and the cranial neuromasts (Figure 1G). Additionally, the primary axon bifurcated within the hindbrain to extend a central process through the spinal cord and a peripheral process that innervated neuromasts of the lateral line (Figures 1F and 1H). Thus, intensely fluorescent, large-diameter neurons located in DC2 of *ETvmat2:GFP* larvae belong to a class of DDNs that innervate the spinal cord and sensory structures of the head and trunk (Figures 1I and 1J).

### Endogenous Activity Patterns

**Figure 2:**
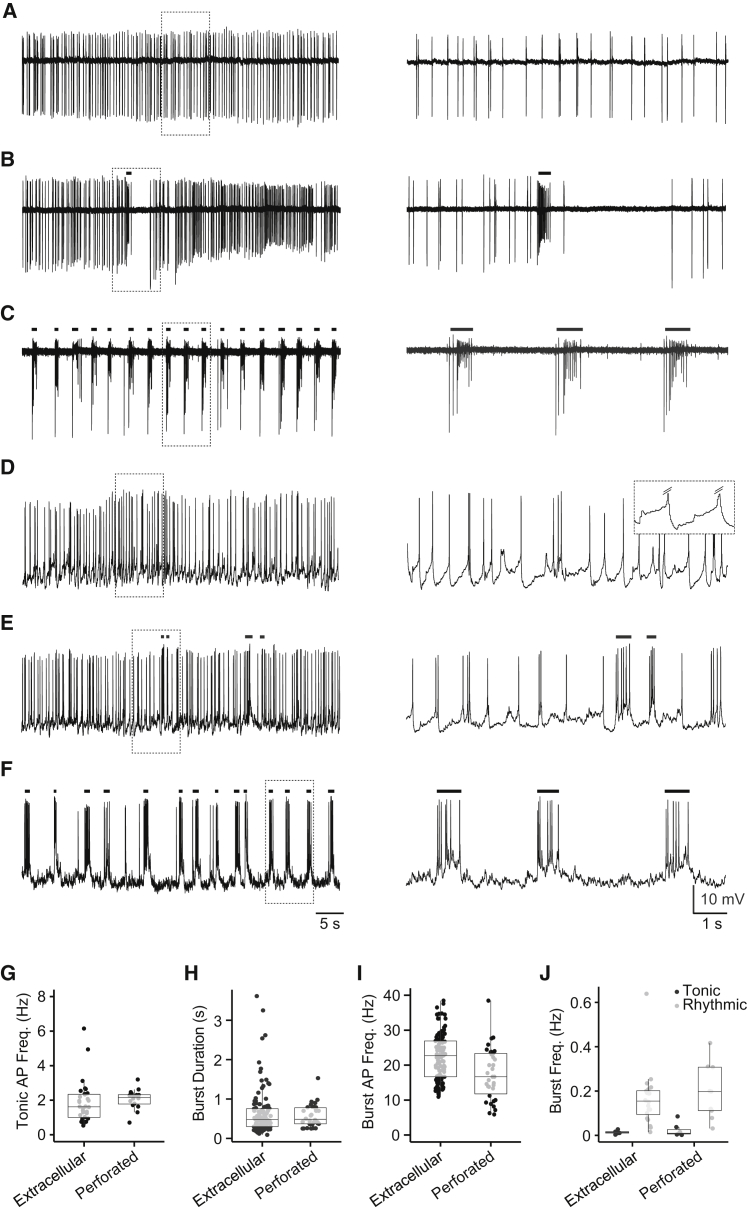
Endogenous DDN Activity Patterns (A–F) Left: representative loose patch (A–C) and perforated patch clamp (D–F) activity patterns recorded from GFP-positive DDNs of awake, paralyzed larvae at 4 dpf. Right: excerpts of activity demarcated by dashed boxes in left panels shown on an expanded timescale. Inset in (D) shows underlying slow membrane oscillations during tonic spiking. Action potentials are truncated (hatched lines). Traces in (A)–(F) are derived from different preparations. (G–J) Box-and-whisker plots of tonic action potential (AP) frequency (G), burst duration (H) within burst AP frequency (I), and the frequency of bursting activity (J) obtained during loose and perforated patch recordings in cells that exhibited primarily tonic spiking (black) or repetitive bouts of bursting (gray). In (G)–(J), filled circles depict raw data points; upper and lower hinges of the box correspond to the first and third quartiles; whiskers extend to 1.5× the interquartile range; and lines within boxes represent median. Scale bars for time in panels (A)–(F) are illustrated in (F). Scale bars for voltage in panels (D)–(F) are illustrated in (F). See also Figure S1.

We next sought to characterize in vivo activity patterns of DDNs recorded from awake larvae paralyzed with the neuromuscular blocker d-tubocurarine (see Supplemental Experimental Procedures). We began by studying spike discharge patterns during loose patch recordings, which permitted non-invasive monitoring of spike activity (see Supplemental Experimental Procedures). All recorded neurons (n = 36) generated tonic discharge patterns that comprised sustained periods of irregular, low-frequency (1.85 ± 0.21 Hz; Figures 2A and 2G) spiking. In 78% of these neurons (n = 28 of 36), tonic spiking was interrupted by bursts of short-duration (0.65 ± 0.05 s; Figures 2B and 2H), high-frequency (23.03 ± 0.77 Hz; Figure 2I) spike discharge. Bursts occurred as either isolated events (n = 8 of 28 neurons; mean burst frequency = 0.02 ± 0.002 Hz; Figures 2B and 2J) or repetitive, rhythmic events (n = 20 of 28 neurons; mean burst frequency = 0.17 ± 0.03 Hz; Figures 2C and 2J). In both cases, bursts were typically followed by a period of silence (mean duration = 2.63 ± 0.29 s) where spike activity was not observed (Figures 2B and 2C). Following these quiescent periods, spike activity recovered, and tonic (Figure 2B) or burst (Figure 2C) firing resumed.

To determine whether tonic and burst modes were coordinated between DDNs, we performed paired loose patch recordings from ipsilateral (n = 8) and contralateral (n = 3) DDN pairs (Figures S1A–S1C). In ipsilateral recordings, 98% (n = 324 of 332) of bursts occurred coincidentally between cells, with a mean firing delay of 11.01 ± 1.35 ms (Figures S1D and S1E). Similar observations were made during contralateral DDN recordings, with 93% of bursts (n = 100 of 108) occurring coincidentally with one another, although mean firing delay increased in these cell pairs (58.18 ± 11.04 ms, p < 0.001, Mann-Whitney U test; Figures S1D and S1E). Periods of tonic spiking also occurred simultaneously in recorded cell pairs, although individual spikes (Figure S1C) were not highly synchronized between ipsilateral (24.9% ± 5.4%) and contralateral (12.2% ± 9.9%) cells (Figures S1C and S1F). These data suggest that tonic and burst firing is coordinated between DDNs within both hemispheres of the brain.

We next used patch clamping to examine the cellular basis of DDN spike patterns. Recordings were made in the perforated patch clamp mode to ensure maintenance of cytoplasmic integrity during experiments (see Supplemental Experimental Procedures). During voltage recordings (n = 21), tonic spiking occurred at a frequency of 2.07 ± 0.14 Hz (Figures 2D and 2G) and appeared to be driven by a combination of spontaneous synaptic activity (Figure 2D) and low-frequency membrane oscillations that were clearly apparent during periods of reduced synaptic input (see inset in Figure 2D). Consistent with loose patch recordings, bursting occurred as either isolated events (7 of 21 neurons, burst frequency = 0.02 ± 0.01 Hz; Figures 2E and 2J) or rhythmic bouts (10 of 21 neurons, burst frequency = 0.22 ± 0.04 Hz; Figures 2F and 2J). In both cases, bursts appeared to arise from powerful depolarizing inputs that were characterized by brief (0.61 ± 0.07 s; Figures 2E, 2F, and 2H) trains of high-frequency (17.45 ± 1.27 Hz; Figure 2I) spike discharge.

### Synaptic Inputs

**Figure 3:**
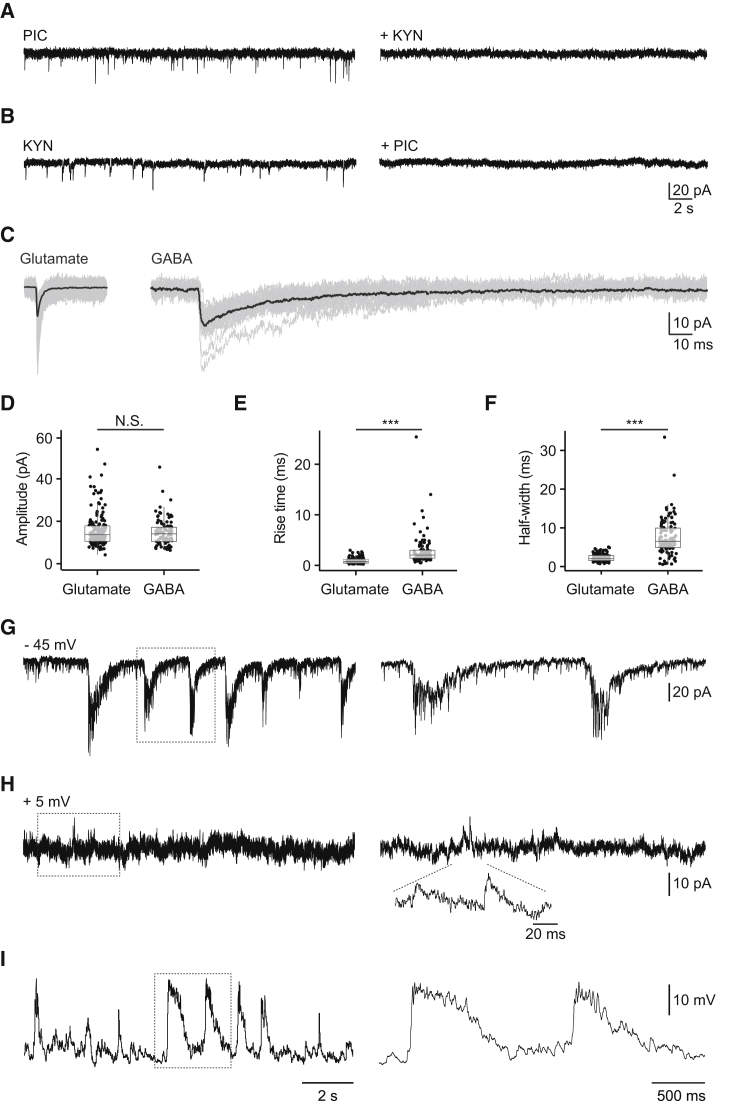
DDNs Receive Glutamatergic and GABAergic Inputs (A) Addition of picrotoxin (PIC) during whole-cell DDN recordings of TTX-treated fish isolated a population of events (left) that were abolished by application of kynurenic acid (KYN; right). (B) Addition of KYN isolated a second population of events (left) that were abolished by addition of PIC (right). (C) Overlays of glutamatergic currents isolated with picrotoxin and GABAergic currents isolated with kynurenic acid (gray traces). Black traces represent current averages. (D–F) Box-and-whisker plots of glutamatergic and GABAergic mPSC amplitudes (D), rise times (E), and half-widths (F). In (D)–(F), filled circles depict raw data points; upper and lower hinges of the box correspond to the first and third quartiles; whiskers extend to 1.5× the interquartile range; and lines within boxes represent median. (G and H) Whole-cell voltage clamp recordings of endogenous synaptic currents recorded from a QX-314-dialyzed DDN of awake, paralyzed larvae clamped at the reversal potential for chloride (G) or cationic (H) currents. (I) Voltage recording derived from the same cell depicting tonic and burst activity. Note that action potentials were blocked in the recorded cell by addition of QX-314 to the pipette solution. Right panels in (G)–(I) are excerpts (dashed boxes) of activity shown on an expanded timescale. Scale bars for time in (G)–(I) are shown in (I).

To determine the nature of synaptic input to DDNs, we exposed preparations to 1 μM tetrodotoxin (TTX), a voltage-gated Na^+^ channel blocker. Under these conditions, spike-dependent transmission is abolished, revealing the presence of miniature postsynaptic currents (mPSCs) that occur as a consequence of spontaneous vesicular exocytosis. The kinetics and pharmacology of mPSCs can be used to derive information about the nature of synaptic input to the recorded cell. Whole-cell recordings obtained using a CsCl-based pipette solution (see Supplemental Experimental Procedures) revealed the presence of two distinct mPSC populations that could be separated on the basis of sensitivity to synaptic blockers. Addition of picrotoxin (100 μM; n = 3), a GABA_A_ receptor antagonist, isolated a population of mPSCs that were likely to be glutamatergic because they were blocked by the pan-specific glutamate receptor antagonist kynurenic acid (2–4 mM; n = 3; Figures 3A and 3C). Conversely, bath application of kynurenic acid (2–4 mM) isolated a second population of events (n = 6; Figures 3B and 3C). These appeared to be mediated by GABA_A_ receptors because they were blocked by the GABA_A_ receptor antagonists picrotoxin (100–200 μM, n = 3; Figure 3B) or bicuculline (25–50 μM, n = 3; data not shown).

In zebrafish and other species, glutamatergic and GABAergic mPSCs have different kinetic properties (R.R. Buss et al., 1999, Soc. Neurosci., abstract). Analysis of mPSCs recorded from DDNs revealed that, as expected, glutamatergic events had faster kinetics than GABAergic events. Specifically, glutamatergic mPSCs had a 10%–90% rise time of 0.86 ± 0.04 ms, a half-width of 2.25 ± 0.07 ms, and an amplitude of 15.68 ± 0.63 pA. GABAergic events had a similar amplitude of 14.72 ± 0.63 pA (p > 0.05, Student’s t test) but had prolonged rise (10%–90% rise time = 2.86 ± 0.30, p < 0.001, Mann-Whitney U test) and decay (half-width = 7.63 ± 0.47 ms, p < 0.001, Mann-Whitney U test) times (Figures 3D–3F). In sum, these observations strongly suggest that DDNs are innervated by glutamatergic and GABAergic inputs.

To determine how these transmitter systems underpin tonic and burst firing, whole-cell voltage clamp recordings were obtained from DDNs in awake d-tubocurarine paralyzed larvae (n = 6). Neurons were voltage clamped at the reversal potential for chloride (approximately −45 mV) and cation-mediated currents (approximately 5 mV) to isolate presumed glutamatergic and GABAergic inputs, respectively. Additionally, QX-314 (2 mM) was added to the patch pipette solution to block Na^+^ channels in the recorded cell, thereby inhibiting action currents, which may mask synaptic inputs. When clamping at the chloride reversal potential, presumed glutamatergic events presented as either irregular synaptic currents or large-amplitude compound currents (Figure 3G). The latter of these are likely to drive bursting because they occurred at a similar frequency (current clamp = 0.30 ± 0.06 Hz, voltage clamp = 0.36 ± 0.07 Hz, p > 0.05, Mann-Whitney U) and had a similar duration (current clamp = 0.49 ± 0.02 s, voltage clamp = 0.41 ± 0.06 s, p > 0.05, Mann-Whitney U) to phasic depolarizations observed in current clamp experiments (cf. Figures 3G and 3I). By contrast, when clamping at the cationic reversal potential, presumed GABAergic currents were sparse and irregular (Figure 3H). When considered in light of our current clamp data, these findings suggest that compound glutamate release drives bursting while irregular glutamatergic and GABAergic release contributes to generation of tonic firing.

### DDNs Spike Autonomously

**Figure 4:**
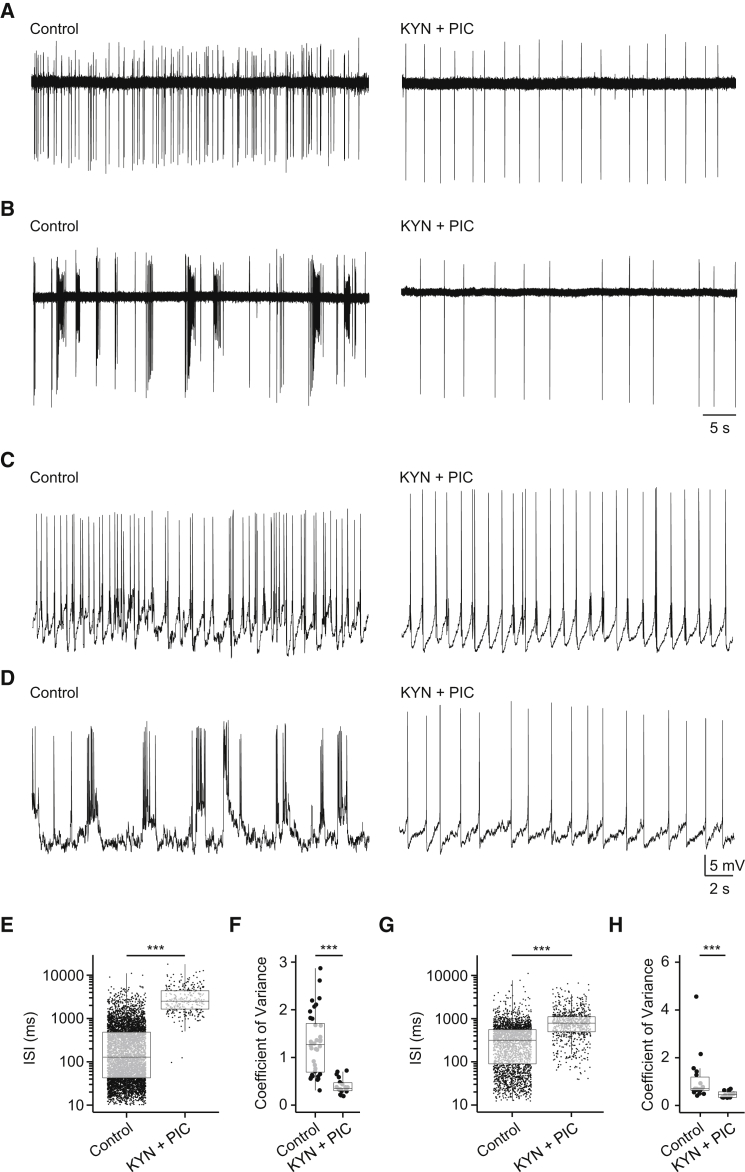
Autonomous Spike Activity in DDNs (A–D) Left: extracellular loose (A and B) and perforated (C and D) patch clamp recordings of DDN activity in awake, paralyzed larvae bathed in control saline. Traces in (A)–(D) are derived from different DDNs. Right: effects of kynurenic acid (2–4 mM) and picrotoxin (50–100 μM) on DDNs that exhibit tonic and intermittent burst spiking (A and C) or repetitive burst spiking (B and D). Note that addition of these blockers unmasks low-frequency autonomous spiking activity. (E–H) Box-and-whisker plots showing the effects of synaptic blockers on interspike interval (ISI; log scaled) and coefficient of variation in loose (E and F) and perforated (G and H) patch clamp recordings. In (E)–(H), filled circles depict raw data points; upper and lower hinges of the box correspond to the first and third quartiles; whiskers extend to 1.5× the interquartile range; and lines within boxes represent median. Scale bars of (A) and (B) are shown in (B); scale bars of (C) and (D) are shown in (D). See also Figure S2.

As membrane oscillations were sometimes apparent during tonic spiking (Figure 2D), we asked whether DDNs possess autonomous spike properties, a feature commonly observed in mammalian DAergic neurons [21]. Cells recorded with the loose patch method (n = 17) were bathed in the synaptic blockers kynurenic acid (2–4 mM) and picrotoxin (50–100 μM). These drugs completely abolished bursting but failed to abolish tonic spiking (Figures 4A and 4B). When compared to recordings conducted in control saline, neurotransmitter-independent spiking occurred at a lower frequency, as reflected by an increase in the interspike interval (ISI; control ISI = 376.89 ± 8.30 ms, synaptic blockers ISI = 3285.6 ± 135.29 ms, p < 0.001, Mann-Whitney U test; Figure 4E), and was more regular, as reflected by a marked decrease in the coefficient of variation for the ISI (control ISI = 1.30 ± 0.10, synaptic blockers ISI = 0.4 ± 0.04, p < 0.001, Mann-Whitney U test; Figure 4F).

Perforated patch clamp recordings (n = 26) confirmed that DDNs spike in the absence of synaptic signaling. Here, co-application of kynurenic acid and picrotoxin abolished synaptic input and burst discharges without altering mean resting potential (control = −55.19 ± 1.28 mV, synaptic blockers = −57.83 ± 1.41 mV, p > 0.05, Student’s t test). However, repetitive spiking persisted in the presence of these drugs (Figures 4C and 4D). Again, spiking was lower in frequency (control ISI = 401.25 ± 9.90 ms, synaptic blockers ISI = 956.18 ± 28.93 ms, p < 0.001, Mann-Whitney U test; Figure 4G) and more regular (ISI coefficient of variation: 1.07 ± 0.23 in control saline and 0.47 ± 0.04 in synaptic blockers, p < 0.001, Mann-Whitney U test; Figure 4H) than activity in control saline. Thus, we conclude that DDNs generate low-frequency autonomous spiking when synaptic transmission is blocked.

Autonomous spikes were superimposed on subthreshold membrane oscillations that resembled oscillatory activity sometimes observed in control saline (cf. Figure S2A and inset in Figure 2D). If these events were driven by voltage-dependent conductances, oscillation frequency should vary as a function of membrane potential. To determine whether this was the case, we injected holding current into DDNs recorded in the perforated patch configuration. Injection of hyperpolarizing current reduced the frequency of oscillations and completely silenced these events at membrane potentials negative to −64.67 ± 3.71 mV (n = 9; Figures S2B–S2E). Termination of the hyperpolarizing current command also caused a transient rebound in autonomous spike frequency (Figures S2C and S2F). By contrast, depolarizing current increased the frequency of oscillations and spike discharges, often evoking doublet or triplet spikes during a single oscillatory cycle (Figures S2B and S2C) while release of depolarizing current transiently decreased action potential frequency (Figures S2C and S2F). These findings are consistent with the hypothesis that autonomous spiking is driven by voltage-dependent conductances.

### Relationship between DDN Activity and Motor Behavior

**Figure 5:**
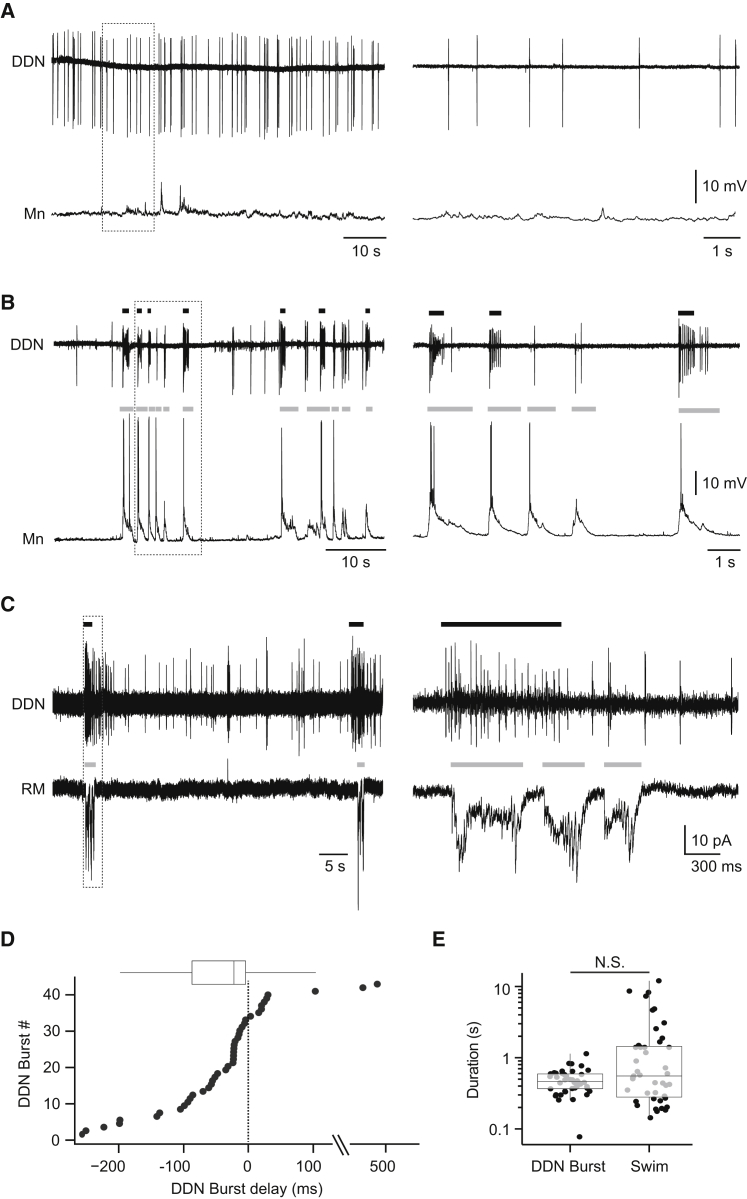
Paired Recordings Reveal the Relationship between Spinal Motor Circuit State and DDN Spike Activity (A and B) Simultaneous loose patch DDN and motoneuron whole-cell recordings obtained from awake, paralyzed larvae at 4 dpf. (A) During periods of motor network inactivity, motoneurons (Mn) do not receive rhythmic locomotor drive, and DDNs spike tonically. (B) During episodes of fictive beat-glide swimming, Mn receive repetitive bouts of locomotor drive during beat periods (denoted by gray bars in lower trace) that often occur simultaneously with DDN bursts (denoted by black bars on upper trace). (C) Simultaneous loose patch DDN and whole-cell red muscle (RM) fiber recording show that DDN bursting (denoted by black bars in upper trace) coincides with rhythmic bouts of locomotor drive to the muscle (denoted by gray bars in lower trace). (D) Plot of delay in onset of DDN burst activity relative to motor episode onset (bottom) with box-and-whisker plot of same data (top). Motor episode occurs at 0 ms (dotted line). (E) Log-scaled duration of DDN bursts and beat episodes. In (D) and (E), filled circles depict raw data points; upper and lower hinges of the box correspond to the first and third quartiles; whiskers extend to 1.5× the interquartile range; and lines within boxes represent median. See also Figure S3.

Although it is widely assumed that DDNs modulate neural encoding of motor activity, the relationship between DDN firing and motor output has not been investigated. We therefore sought to define the behavioral contexts associated with tonic and burst DDN discharges. To do this, we performed simultaneous recordings of DDNs (using the loose patch configuration) and spinal motoneurons or muscle fibers (using the whole-cell configuration; n = 13). At larval stages, zebrafish exhibit spontaneous locomotor activity that comprises alternating periods of tail beating that are separated by short glide periods. In paralyzed larvae, the fictive correlate of this behavior, termed “beat-glide” swimming, presents as alternating bouts of rhythmic synaptic drive separated by short silent periods where the membrane potential returns to rest [22]. Simultaneous DDN-motoneuron or muscle fiber recordings revealed that tonic spike activity occurred when animals were not engaged in locomotor behavior (Figures 5A and 5C). By contrast, burst discharges were rarely observed at rest: of the 45 burst discharges observed across all experiments, only two occurred during periods of inactivity (data not shown). However, the incidence of bursting markedly increased when fish engaged in fictive locomotion. Specifically, 96% (n = 43 of 45) of bursts occurred contemporaneously with the beat component of swimming (Figures 5B and 5C). Nonetheless, a notable proportion of beat episodes (39%, n = 28 of 71) were not accompanied by bursts (Figures 5B and 5C). Similarly, the duration of beat episodes (mean beat duration in absence of DDN burst = 1.53 ± 0.5 s, mean beat duration in presence of DDN burst = 1.57 ± 0.44 s, p > 0.05, Mann-Whitney U test; Figure S3A) or the following rest period (mean rest period in absence of DDN burst = 20.3 ± 10.7 s, mean rest period in presence of DDN burst = 16.30 ± 3.44 s, p > 0.05, Mann-Whitney U test; Figure S3B) was not affected by the presence of DDN bursts. This suggests that DDNs do not regulate the basic pattern of beat-glide swimming activity.

Examination of the delay between DDN burst onset and beat episode onset revealed that 77% of DDN bursts (n = 33 of 43) occurred before (mean delay = 74.92 ± 12.76 ms) and 23% (n = 10 of 43) occurred after (mean delay = 119.70 ± 60.01 ms) onset of motor activity (Figure 5D). Additionally, the duration of DDN bursts was, on average, less variable but not significantly different from beat episodes (DDN burst duration = 488.5 ± 28.56 ms, beat episode duration = 1710 ± 406.8 ms, p > 0.05, Mann-Whitney U test; Figure 5E). Thus, although bursting is strongly correlated with motor output, it is neither necessary nor sufficient for initiation of locomotion.

### DDN Ablation Affects Motor Behavior

**Figure 6:**
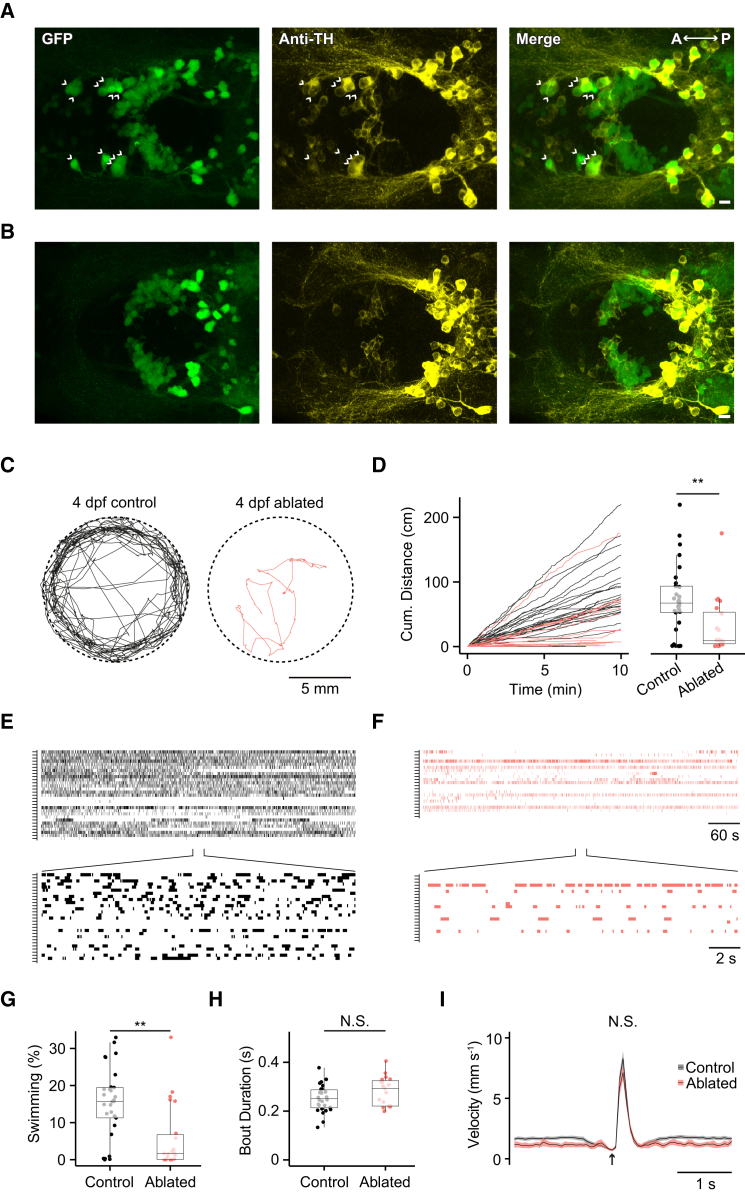
Effects of DDN Ablation on Zebrafish Behavior (A and B) Ventral views of GFP (left), anti-tyrosine hydroxylase (anti-TH; middle), and merged GFP/anti-TH (right) labeling in the diencephalon of control (A) and ablated (B) *ETvmat2:GFP* larvae at 4 dpf. Note that the anterior-most neurons corresponding to DDNs in DC2 (arrowheads in A) are absent in ablated fish. (C) Swimming trajectories of 4 dpf control and laser ablated zebrafish recorded over a 10 min period. (D) Left: cumulative distance that individual control (black) and laser-ablated (red) fish travel over the 10 min period. Right: box-and-whisker plot of total distance traveled in 10 min period for control and laser-ablated 4 dpf larvae. (E and F) Raster plots of identified episodes of beat-glide swimming in non-ablated (E) and ablated (F) 4 dpf larvae over the 10 min recording (top). Bottom: corresponding regions of raster plots over an expanded timescale. Each row represents a single fish during the recording. (G and H) Box-and-whisker plots of the percent of time spent beat-glide swimming over the 10 min observation period (G) and the duration of individual beat-glide bouts (H). (I) Plots of average velocity as a function of time for bouts of beat-glide activity. At the onset of the beat period (black arrow), velocity increases sharply and subsequently declines to baseline levels during the glide period. For box-and-whisker plots in (D), (G), and (H), filled circles depict raw data points; upper and lower hinges of the box correspond to the first and third quartiles; whiskers extend to 1.5× the interquartile range; and lines within boxes represent median. Scale bars of (A) and (B) represent 10 μm; anterior is left, posterior is right. See also Figures S4 and S5.

Since DDN bursting coincided with locomotor output, we next asked whether abrogation of supraspinal DA signaling affected expression of locomotor behavior. Previous pharmacological, genetic ablation and lesion studies have suggested that manipulation of DDN activity perturbs zebrafish expression of beat-glide swimming activity [13]. However, these interventions may also affect other DAergic and non-DAergic cell populations, some of which may be involved in control of motor output. We therefore sought a more specific method for disrupting spinal DA signaling. DDNs are the earliest developing DAergic neurons of the zebrafish diencephalon, first forming at around 16 hours post fertilization (hpf) [19, 20]. As such, we asked whether laser-ablating GFP-positive neurons at 1 dpf caused selective loss of DDNs at larval stages. Since DC2 neurons were the focus of our physiological studies, we first attempted to ablate this cell group. To do this, we targeted GFP-positive neurons in the anterior aspect of the posterior tuberculum of 20–24 hpf embryos for laser ablation (Figures S4A and S4B). Subsequently, analysis of GFP and TH labeling at 4 dpf (n = 10) revealed a marked reduction in the number of large-diameter labeled cells in DC2 of the posterior tuberculum (control = 10.5 ± 0.37, n = 12; ablated = 1.18 ± 0.48, n = 11; p < 0.001, Mann-Whitney U test; Figures 6A and 6B) while surrounding GFP-positive cells appeared unaffected by this treatment. This suggests that ablation of large-diameter, anteriorly located GFP-positive neurons in the embryo causes selective loss of DDNs in DC2 of zebrafish larvae.

We next examined motor activity in ablated fish. During behavioral analysis, both control (28 of 29) and ablated (19 of 22) larvae exhibited bouts of beat-glide swimming that were characterized by alternating periods of motor activity and quiescence (Figures 6C–6F). However, we observed marked differences in total distance swam. Specifically, non-ablated larvae covered 72.95 ± 9.92 cm (n = 29) over a 10 min period while ablated fish covered only 30.37 ± 9.71 cm (n = 22, p < 0.01, Mann-Whitney U test; Figures 6C–6F). This effect was clearly apparent in raster plots of individual beat-glide episodes plotted as a function of time (Figures 6E and 6F). Further analysis revealed that these changes were caused by a decrease in the proportion of time spent swimming: on average, control larvae were engaged in beat-glide swimming for 15.34% ± 1.75% of the 10 min period while ablated larvae were active for only 5.81% ± 1.86% of this time period (p < 0.01, Mann-Whitney U test; Figure 6G). However, the mean duration (control = 0.25 ± 0.01 s, ablated = 0.28 ± 0.01 s, p > 0.05, Mann-Whitney U test; Figure 6H) and mean peak velocity (control = 8.35 ± 0.59 mm s^−1^, ablated = 7.6 ± 0.64 mm s^−1^, p > 0.05, Mann-Whitney U test; Figure 6I) of beat-glide episodes were not affected.

We also tested the effects of ablating all large-diameter GFP-positive neurons in the posterior tuberculum of *ETvmat2:GFP* embryos (30–32 hpf). Examination of this treatment group at larval stages revealed a loss of large-diameter neurons in DC2, DC4, and DC5 of the posterior tuberculum (n = 12; Figures S5A and S5B). As a proportion of DC4/5 neurons also project to the spinal cord [15], we used these fish to examine the behavioral consequences of more widespread DDN ablation. We found that motor effects were similar to those observed in DC2 ablated fish. Specifically, episodes of beat-glide swimming were of a similar duration (control = 0.29 ± 0.01 s, DC2 and DC4/5 ablated = 0.26 ± 0.03 s, p > 0.05, Mann-Whitney U; Figure S5H) and velocity (control = 8.35 ± 0.64 mm s^−1^, DC2 and DC4/5 ablated = 6.74 ± 1.14 mm s^−1^, p > 0.05, Student’s t test) to controls (Figure S5I). However, the incidence of beat-glide episodes was dramatically reduced (Figures S5E and S5F), as reflected by a decrease in the percentage of time spent swimming (control = 13.5% ± 2.14%, ablated = 0.84% ± 0.34%, p < 0.001, Mann-Whitney U, Figure S4G) and distance covered (control = 55.75 ± 10.61 cm, ablated = 2.27 ± 1.00 cm, p < 0.001, Mann-Whitney U; Figure S4D). Thus, widespread ablation of large-diameter DAergic neurons in DC2 and DC4/5 suppresses motor activity without affecting motor patterning.

## Discussion

In this study, we have examined the in vivo activity patterns and functional properties of DDNs within the larval zebrafish brain. Our main findings are that DDNs generate two forms of firing activity: tonic firing, which correlates with periods of inactivity, and burst firing, which correlates with bouts of locomotor behavior. Additionally, we demonstrate that DDN ablation reduces locomotor activity without affecting patterning of beat-glide swimming. For the first time, to our knowledge, our findings describe the behavioral contexts associated with DDN firing and provide novel insights into the functional relevance of these cells.

### Endogenous DDN Activity Patterns

Loose patch recordings obtained from awake, paralyzed zebrafish revealed that DDNs exhibit two modes of firing: low-frequency (1–2 Hz) tonic spiking and high-frequency (∼20 Hz) bursting, which is followed by a short pause in spike activity. In the large majority of preparations (78%), DDNs alternated between these two modes of firing. The remaining preparations generated purely tonic spike activity, most likely because they did not express locomotor activity during the recording period (see below). These activity patterns mirror those observed during extracellular recordings of mammalian mesodopamine neurons, which exhibit mixed tonic and burst firing modes of activity [23–25]. Thus, our observations suggest DDNs encode information in a manner that is consistent with other DAergic neuron classes of the brain. Additionally, our findings also suggest a high degree of functional conservation between teleostan and mammalian DAergic neurons, thus underscoring the utility of the zebrafish as a model for studying physiological aspects of DA signaling.

As larval zebrafish are accessible to in vivo patch clamp recording, we were able to examine the cellular basis of endogenous DDN activity patterns. Our voltage clamp data show that burst discharges appear to be driven by powerful volleys of glutamate release, whereas tonic spiking is driven by a combination of regular autonomous spiking and irregular glutamate and GABA release, which presumably reduces the regularity of spike discharge. This interpretation is based on the finding that synaptic blockers abolish bursting and transform irregular tonic spiking into regular, low-frequency spiking that is underpinned by membrane oscillations that cyclically drive cells to spike threshold. Our findings help to address a longstanding problem associated with the study of mesodopaminergic pathways: preparations that are typically accessible to intracellular studies (such as cell and tissue culture models) invariably generate low-frequency autonomous spike activity while extracellular recordings of awake-behaving animals exhibit tonic and burst spike activity [21]. It is assumed that these differences arise from a loss of afferent input in preparations that are accessible to intracellular recording, resulting in expression of purely autonomous spike activity. Support for this hypothesis is derived from in vitro dynamic clamp studies, which show that burst firing can be elicited by either introduction of *N*-Methyl-D-aspartic acid (NMDA) or removal of GABAergic conductances [26–28]. However, direct in vivo evidence has been lacking. Our in vivo DDN recordings demonstrate that synaptic input is necessary for expression of tonic and burst firing but that regular, autonomous spike activity is unmasked when synaptic input is disrupted.

Like mammalian mesodopamine neurons [21], autonomous DDN spiking is driven by voltage-dependent membrane oscillations that cyclically drive cells to action potential threshold. Evidence for this comes from the observation that oscillatory frequency is strongly affected by current injection. Although the intrinsic conductances underpinning membrane oscillations have yet to be defined, these findings lend further support to the hypothesis that both DDNs and mesodopamine neurons encode information through convergent physiological mechanisms.

### The Functional Relevance of DDNs

Paired recording experiments show that tonic and burst firing are linked to distinct behavioral states. Specifically, DDNs spike tonically during periods of inactivity and burst synchronously during periods of locomotor output. These observations suggest that spinal neurons are exposed to a basal tone of DA when the animal is at rest but experience large transient increases in DA during bouts of locomotion. Correlations between bursting and locomotor output notwithstanding, two lines of evidence suggest that DDNs are neither necessary nor sufficient for locomotion: first, not all motor episodes are accompanied by bursts; and second, burst onset is variable, sometimes preceding and sometimes proceeding onset of locomotor output. This strongly suggests that, unlike descending DAergic pathways that innervate the lamprey brainstem [29], spinally projecting DDNs do not initiate locomotion.

Our laser ablation studies provide insights into the behavioral significance of the DDN population. We found that targeted ablation of DC2 neurons, which were the subject of our physiological analysis, markedly decreased the total time spent swimming. When DC4/5 neurons were also ablated, we observed a similar yet stronger effect on motor output. Although this suggests that DDNs of DC2 and DC4/5 have equivalent functions, it should be noted that relationships between DC4/5 neuron firing and motor output have yet to be demonstrated. Nonetheless, we found that both treatments did not affect the velocity or duration of individual beat-glide episodes. This finding, which suggests DDNs regulate spinal cord excitability without affecting locomotor patterning, stands in contrast to a previous study showing reversion to embryonic forms of behavior on block of DA (D4 subtype) receptors, genetic deletion of *otpb*-expressing neurons, or forebrain transection [13]. The reasons for this discrepancy remain to be determined. However, one possible explanation is that the transection and genetic ablation techniques previously used to interrogate DDN function may also impact non-DDN cell populations. For example, transections remove all descending inputs while chemogenetic ablation of *otpb*-expressing cells is expected to ablate non-DAergic neuroendocrine cells [30]. Loss of these neurons may contribute to the previously reported disruption of motor patterning, which is not observed following laser ablation of DC2 (or DC2 and DC4/5) neurons.

Another important consideration is that tonic and phasic DA release is likely to have differential effects on the spinal network. Evidence from computational studies of mammalian mesodopamine neurons [31] suggests that DA released under tonic discharge conditions will occupy the majority of high-affinity D2-like receptors, but the lower-affinity D1-like receptors will remain largely unoccupied. Conversely, during phasic discharge, DA concentrations will transiently increase. This will preferentially increase the relative occupancy of D1-like receptors (because at basal levels, D2-like receptors are already approaching saturation). During beat-glide swimming, DDNs undergo repeated cycles of bursting that are separated by periods of silence. These firing patterns resemble burst-pause spike activity seen in mammalian mesodopamine neurons, which are proposed to cause a net decrease in D2-like receptor occupancy and a net increase in D1-like receptor occupancy [31]. In other vertebrates, D1-like receptors facilitate [6, 9, 32–34] and D2-like receptors depress [6] motor network activity, while in zebrafish, D1/D4 receptors facilitate and D2/D3 receptors suppress locomotion [8, 13]. Thus, DA is expected to have complex and dynamic modulatory actions that depend on the temporal nature of DDN firing and the location of spinal D1-like and D2-like receptors. Future optogenetic and targeted genetic ablation methods, such as those recently used in mammalian mesodopamine studies [35, 36], hold great promise for delineating the modulatory functions and behavioral effects of tonic and phasic modes of DA release in the vertebrate locomotor network.

### Possible Sources of Input to the DDN Population

Although sources of input to the posterior tuberculum have yet to be fully described, evidence suggests that this region integrates information from a variety of brain areas. For example, the zebrafish posterior tuberculum receives extensive input from the olfactory bulb [37], and in the lamprey, olfactory signals are relayed through the posterior tuberculum to locomotor-related reticulospinal cells of the hindbrain [38]. The hypothalamic hypocretin (orexin) system provides another source of input to the posterior tuberculum. This system, which has been implicated in controlling arousal, branches extensively within the posterior tuberculum, forming putative synapses with TH-positive neurons in this area [39, 40]. Finally, there is also evidence that the posterior tuberculum is innervated by DAergic neurons of the subpallium [15], a structure considered to be equivalent to the mammalian basal ganglia. Given this, DDNs may modulate spinal network excitability in response to a wide range of behavioral needs. Future anatomical and physiological studies will undoubtedly help to define the neural pathways that drive activity in this forebrain DAergic neuron population.

### DDN Input to the Lateral Line

Our neurobiotin labeling experiments revealed that DDNs also innervate neuromasts of the lateral line. These mechanosensory structures act as distant motion sensors, detecting perturbations in water flow. Thus, DDNs are likely to have dual roles in modulating motor network output and mechanosensory processing. In this context, a possible role for DAergic signaling is in the gating of lateral line sensitivity during motor behavior. Zebrafish are known to exhibit reduced sensitivity to flow perturbations when swimming [41]. Moreover, studies of dogfish and burbot show that the lateral line receives inhibitory efferent input and that this structure is inhibited during locomotion [42, 43]. These effects are likely to be behaviorally relevant, as they will prevent lateral line activation in response to self-induced flow perturbations caused by locomotor activity. Future studies will help to determine whether release of DA from DDNs has a role in mediating this effect.

## Experimental Procedures

Electrophysiology and behavioral experiments were conducted on 4 dpf (96–110 hpf) *ETvmat2:GFP* zebrafish larvae. Animals were maintained according to established procedures [44] and in compliance with the Animals (Scientific Procedures) Act 1986. Adult fish were inbred, and embryos were harvested for incubation at 28.5°C until reaching the required developmental stage. Full experimental details are provided in the Supplemental Experimental Procedures.

## Author Contributions

J.R.M. and M.J. conceived and designed the experiments. M.J., J.R.M., and F.D.F. performed the experiments. M.J., J.R.M., and F.D.F. analyzed the data. J.R.M., M.J., and F.D.F. wrote the paper.

## Supplemental Information

Document S1. Supplemental Experimental Procedures and Figures S1–S5

Document S2. Article plus Supplemental Information
