# Supplementary material for: Firing Dynamics and Modulatory Actions of Supraspinal Dopaminergic Neurons during Zebrafish Locomotor Behavior
Source: Curr Biol. 2015 Feb 16;25(4):435–44. doi: 10.1016/j.cub.2014.12.033 (PMC4331284; doi:10.1016/j.cub.2014.12.033)
Supplement: Document S1. Supplemental Experimental Procedures and Figures S1–S5 [file mmc1.pdf]

Current Biology
Supplemental Information

Firing Dynamics and Modulatory Actions of Supraspinal Dopaminergic Neurons during Zebrafish Locomotor Behavior

Michael Jay, Francesca De Faveri, and Jonathan Robert McDearmid

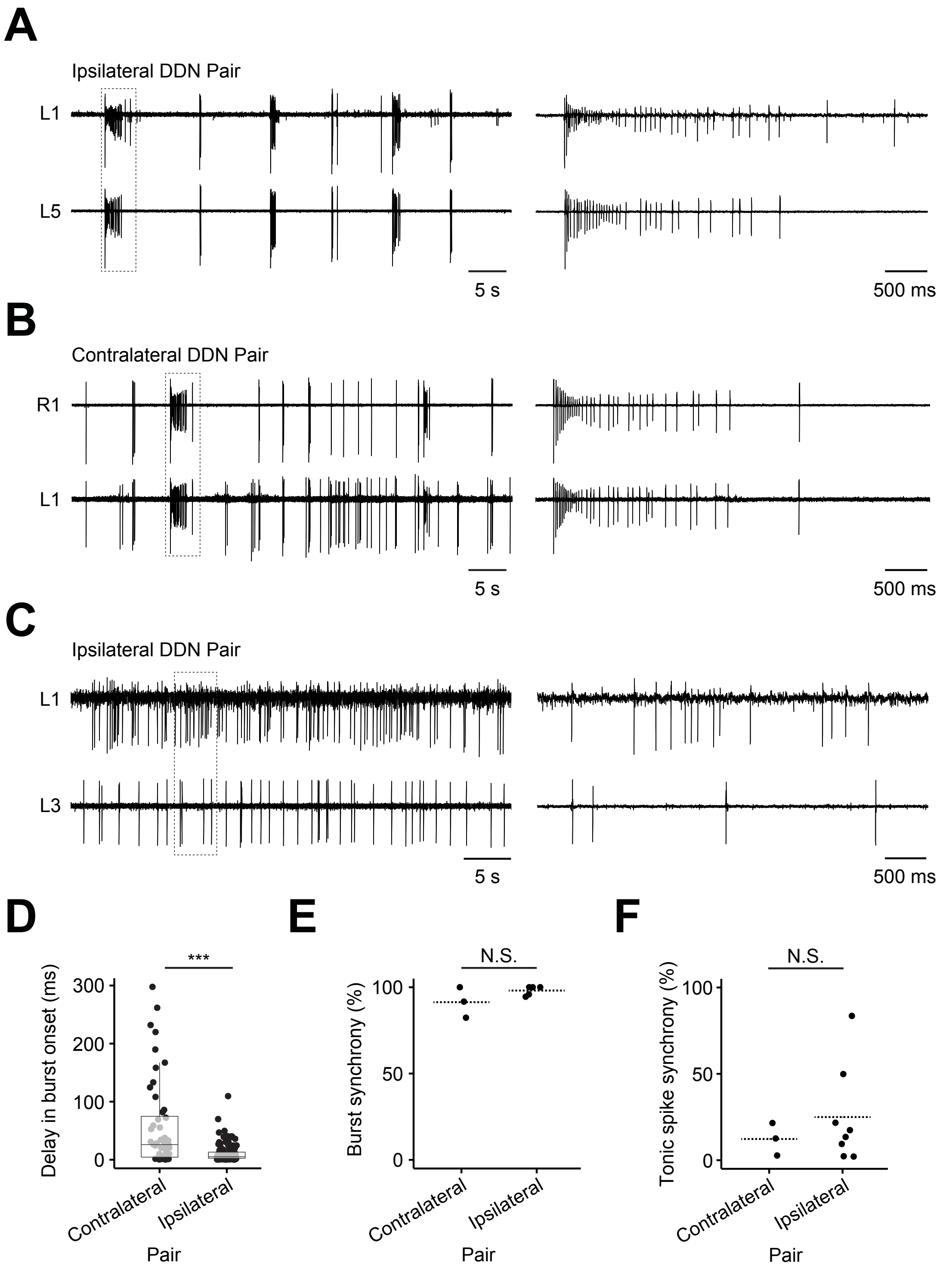

Figure S1: Firing Activity is Coordinated Between DDNs. Related to Figure 2. (A – C) Paired loose patch clamp recordings from ipsilateral (A) and contralateral (B) DDN pairs exhibiting bursting activity and from an ipsilateral DDN pair exhibiting tonic spiking activity (C). Cells are labelled according to the hemisphere in which they were located ('R' = right, 'L' = left) and their relative position within the DDN cluster (1 = most anterior, 5 = fifth most anterior). **(D)** Box and whisker plot showing the delay in burst onset between recorded DDNs. In (D) filled circles depict raw data points; upper and lower hinges of the box correspond to the first and third quartiles; whiskers extend to 1.5 x the interquartile range; and lines within boxes represent median. **(E – F)** Plots of synchrony between bursts (E) and synchrony between tonic spiking during paired recordings (F). In (E – F) filled circles depict raw data points and dotted lines represent means.

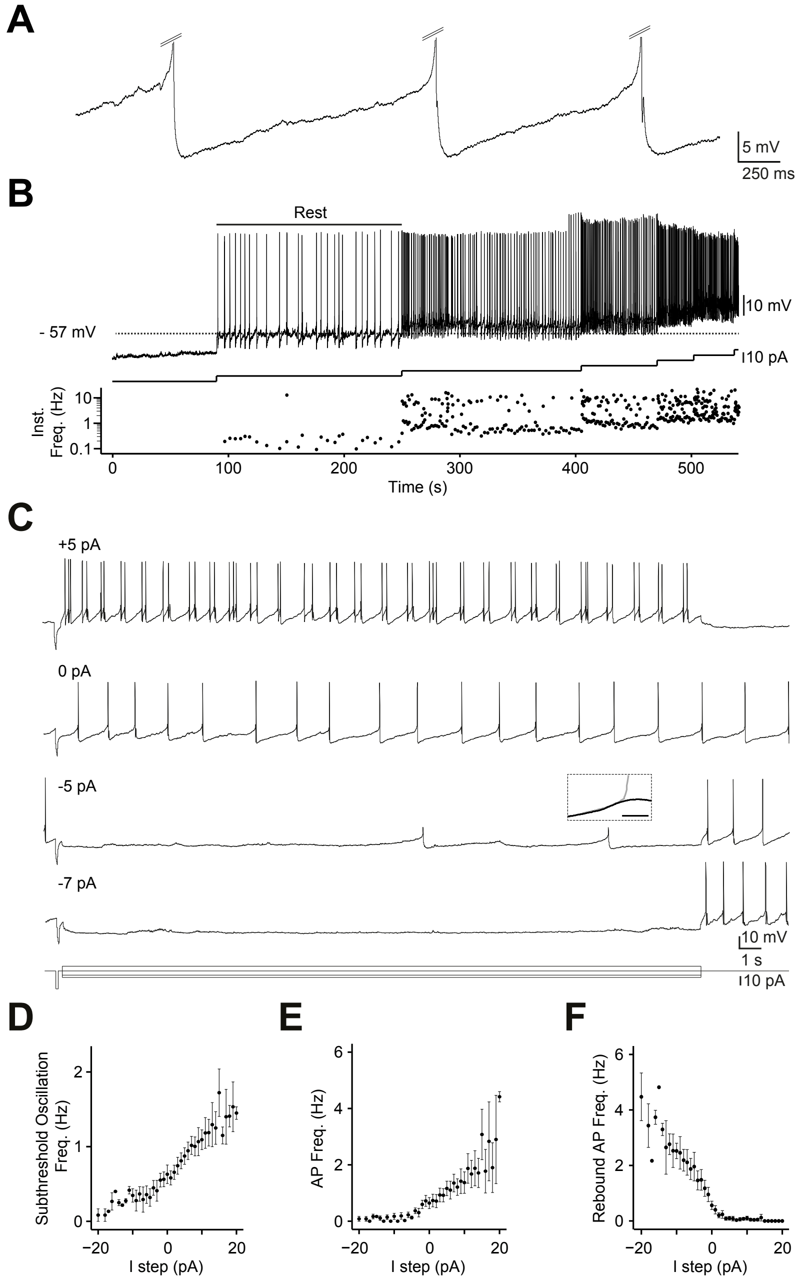

Figure S2: Autonomous Spiking is Voltage-dependent. Related to Figure 4. (A) Magnified region showing subthreshold membrane potential oscillations that underpin autonomous DDN spike discharges in a larvae treated with kynurenic acid (4 mM) and picrotoxin (100 µM). Action potentials in this trace are truncated (hatched lines). **(B)** Upper panel: representative perforated patch clamp recording showing effects of current injection (middle panel) on autonomous spiking in a DDN exposed to kynurenic acid (4 mM) and picrotoxin (100 µM). Lower panel: log scaled plot of instantaneous spike frequency for the recording displayed in the upper panel. Note that spike frequency is strongly dependent on the magnitude of current injection. Black bar in upper panel (Rest) denotes period where no holding current was applied. **(C)** Depolarising current steps increase the frequency of membrane oscillations and autonomous spiking whilst hyperpolarising current steps abolish spiking and slow, or even inhibit, subthreshold oscillations. Release from negative current causes a transient increase in spike frequency. Inset (dashed box): excerpt of activity depicting the rising phase of a membrane oscillation that failed to reach spike threshold (black) superimposed on a membrane oscillation that triggered an action potential (grey). Scale bar in inset is 1 ms. **(D – F)** Plots of mean subthreshold oscillation frequency (D), action potential (AP) discharge frequency (E) and transient rebound AP frequency (F) as a function of current injection magnitude. In (D – F) data are represented as mean ± SEM.

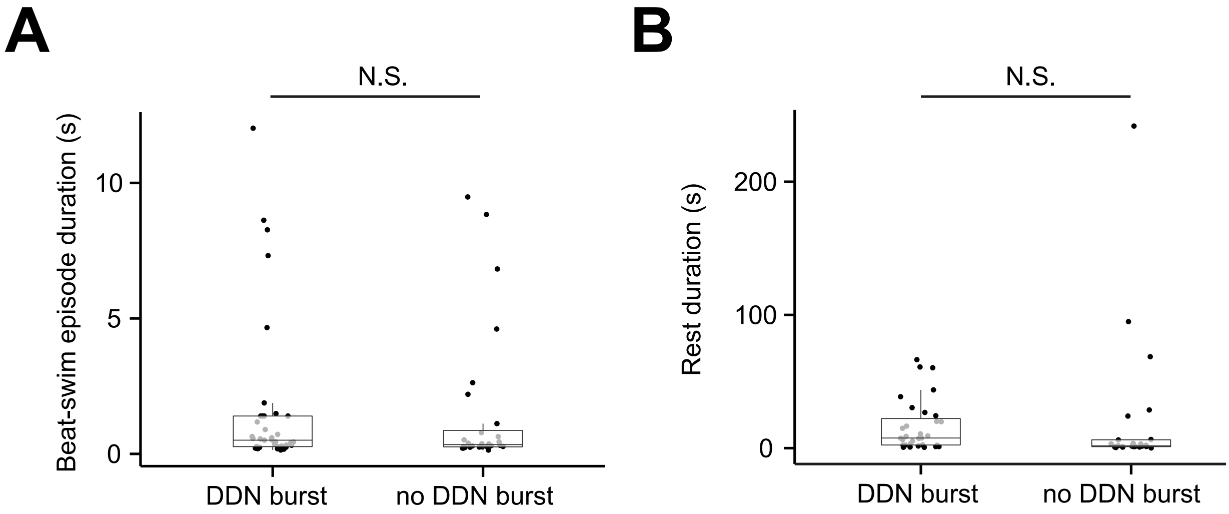

Figure S3: Duration of locomotor activity is unaffected by DDN bursting. Related to Figure 5. (A – B) Motor episodes occurring in the presence or absence of DDN burst activity have similar beat (A) and rest (B) durations. In (A,B) filled circles depict raw data points; upper and lower hinges of the box correspond to the first and third quartiles; whiskers extend to 1.5 x the interquartile range; and lines within boxes represent median.

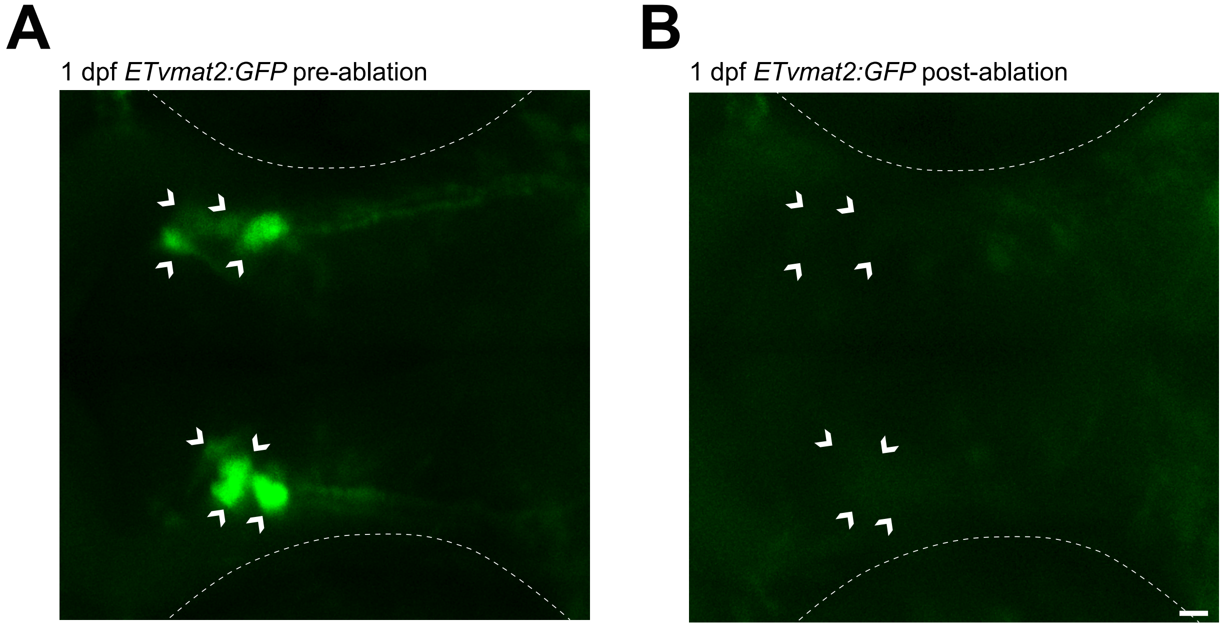

Figure S4: Targeted ablation at 1 dpf causes loss of GFP positive cells. Related to Figure 6. (A – B) GFP positive cells within the diencephalon of a 20 hpf *ETvmat2:GFP* larvae (A) were targeted for ablation. To do this, cells were briefly irradiated with a UV laser which caused the loss of large intensely fluorescent GFP positive cells (B). Arrow heads indicate cells targeted for ablation. White dashed lines mark the eyes. Scale bars (A – B) in (B), 10 µm; anterior is left, posterior is right.

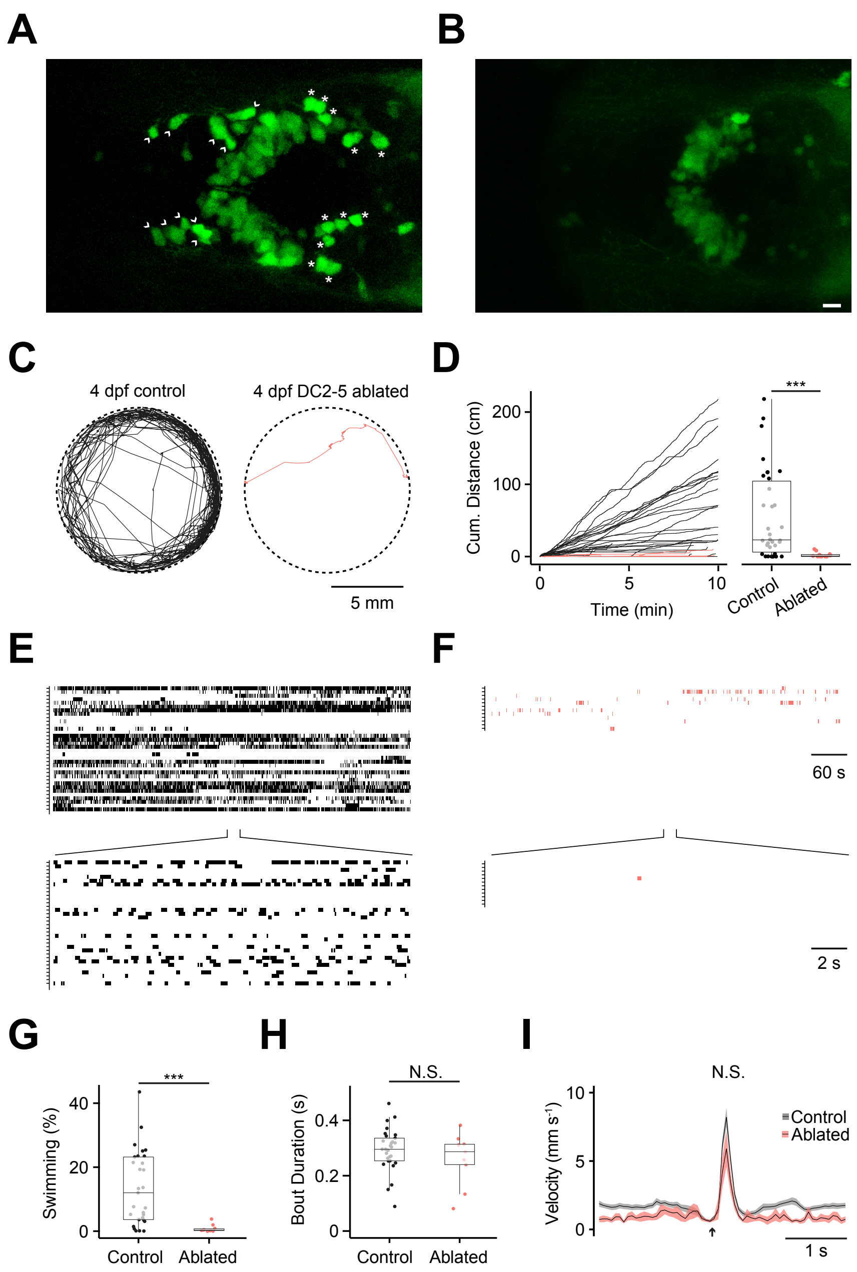

Figure S5: Effects of DC2 and DC4/5 Ablation on Zebrafish Behaviour. Related to Figure 6. (A – B) Ventral images of the diencephalon of control (A) and ablated (B) *ETvmat2:GFP* larvae at 4 dpf. Control fish (A) have a full complement of GFP-expressing cells in DC2 (arrow heads) and DC4/5 (asterisks) whilst laser ablation of all large diameter cells at 30 hpf results in loss of these neurons at 4 dpf (B). **(C)** Swimming trajectories of 4 dpf control and laser ablated zebrafish recorded over a 10 minute period. **(D)** Left: cumulative (cum) distance that individual control (black) and laser ablated (red) fish travel over the 10 minute period. Right: box and whisker plot of total distance travelled in 10 minute period for control and laser ablated 4 dpf larvae. **(E – F)** Raster plots of identified episodes of beat-glide swimming in non-ablated (E) and ablated (F) 4 dpf larvae over the 10 minute recording (top). Bottom panels: corresponding raster plots over an expanded time scale. Each row represents a single fish during the recording. **(G – H)** Box and whisker plots of the percent time spent beat-glide swimming over the 10 minute observation period (G) and the duration of individual beat-glide bouts (H). **(I)** Plots of average velocity as a function of time for bouts of beat-glide activity. At the onset of the beat period (black arrow) velocity increases sharply and subsequently declines to baseline levels during the glide period. Scale bars (A – B) in (B), 10 µm; anterior is left, posterior is right. For box and whisker plots in (D,G,H) filled circles depict raw data points; upper and lower hinges of the box correspond to the first and third quartiles; whiskers extend to 1.5 x the interquartile range; and lines within boxes represent median.

SUPPLEMENTAL EXPERIMENTAL PROCEDURES

Electrophysiology

For all electrophysiology experiments, fish were anaesthetised in MS-222 (0.02 %) dissolved in Evan's physiological saline (composition in mM: 134 NaCl, 2.9 KCl, 2.1 $CaCl_2$, 1.2 $MgCl_2$, and 10 HEPES, pH 7.8) and pinned to a Sylgard-lined Petri dish with 25 µm diameter tungsten wires that were inserted through the notochord. A pair of fine forceps were used to carefully remove one eye so that the underlying diencephalon was exposed. The preparation was then transferred to a patch clamp setup and perfused with Evan's physiological saline containing glucose (10 mM). To study endogenous activity patterns in awake larvae, d-tubocurarine (10 µM, Sigma) was added to the extracellular saline. By including this neuromuscular blocker we were able to prevent muscle contractions that would normally disrupt electrophysiological recordings, thereby permitting the study of nervous system activity in awake, unanaesthetised animals. For recordings of miniature postsynaptic currents (mPSC), the voltage-gated Na^+ channel blocker tetrodotoxin (1 µM, Ascent Scientific) was added to the extracellular saline, thereby blocking voltage gated Na^+ channels to unmask quantal neurotransmitter release. Additionally, in mPSC experiments a low Mg^{2+} Evan's physiological saline (composition in mM: 134 NaCl, 2.9 KCl, 3.3 $CaCl_2$ and 10 HEPES, pH 7.8) was used to alleviate block of NMDA receptors.

Patch clamp electrodes (resistance = 3 – 10 MΩ) were pulled from borosilicate glass (Harvard Apparatus, UK) using a P-80 micropipette puller (Sutter Instrument, USA). For loose patch recordings, electrodes filled with Evan's extracellular saline were used to obtain low resistance (15 – 25 MΩ) seals that enabled non-invasive observation of extracellular spike discharges. Loose patch methods were also used for juxtacellular labelling experiments. However in this case, 0.5 % neurobiotin (Vector Labs) was included in the pipette solution and repeated 0.5 Hz, 500 ms depolarising current commands (approximately 1 nA) were applied for 30 – 45 min to facilitate transfer of neurobiotin to the cell.

For whole cell recordings of motoneurons and muscle fibres, patch electrodes were filled with a K-gluconate based solution containing (in mM): 10 HEPES, 10 EGTA, 2 $MgCl{\cdot}6H_2O$, 10 NaCl, 6 KCl and 126 D-gluconic acid-potassium salt (pH adjusted to 7.2 with KOH). Whole cell methods were also used for voltage clamp recording of mPSCs. In this case, a CsCl based electrode solution containing (in mM): 10 HEPES, 10 EGTA, 2$MgCl{\cdot}6H_2O$ and 135 CsCl was used. With this solution, the reversal potential for Cl^- ions was strongly depolarised such that GABAergic events could be readily resolved as large inward currents when clamping at negative membrane potentials (holding potential for mPSC experiments was -75 mV). In addition, whole cell voltage clamping was also used to study endogenous synaptic drive to DDNs. In these experiments, QX-314 bromide (2 mM, Tocris) was added to the intracellular K-gluconate solution to prevent initiation of action currents at holding potentials positive to action potential threshold. Here, the reversal potential of cationic and chloride currents was experimentally determined by observing the reversal potential of spontaneous synaptic currents.

For current clamp recordings of endogenous DDN activity, perforated patch clamp methods were used. We chose this approach because we observed gradual washout of autonomous spiking when in the whole cell configuration. Here, amphotericin B was dissolved in DMSO to a final concentration of 1 mg ml^{-1}. The stock solution was subsequently diluted in K-gluconate to a final concentration of 10 µg ml^{-1}. Amphotericin B stock solutions were discarded and replaced with fresh solution after 12 hours use.

The fluorescent features of the patch clamp microscope (Nikon FN-1) were used to target GFP-labelled neurons of *ETvmat2:GFP* fish for electrophysiological recording. During whole-cell experiments, sulforhodamine B (0.1 %) was routinely added to the patch pipette solution so that projection patterns of recorded neurons could be examined using fluorescence microscopy. Similar approaches were used for perforated patch experiments, although in these instances the patch was ruptured after completion of experiments to facilitate dialysis of the recorded cell. Cells that lacked spinally projecting axons were excluded from analysis.

Recordings were amplified using a Multiclamp 700B (Molecular Devices, Sunnyvale, CA, USA) amplifier. Data were digitised with an Axon Digidata 1440A (Molecular Devices, Sunnyvale, CA, USA) A-D converter connected to a PC running pClamp 10 (Molecular Devices). Raw signals were acquired at 10 - 20 kHz. During whole cell recordings, signals were low-pass filtered at 10 kHz and during extracellular loose patch

recordings signals were band pass filtered between 1 and 4 kHz.

Electrophysiological analysis was conducted offline using Clampfit (Molecular Devices). For analysis of mPSCs, a template matching function was used to isolate populations of events. For both extra- and intracellular recordings of endogenous activity, events were detected using a threshold detection function. During analysis of bursting activity, bursts were defined as discrete periods of spiking that lasted for 150 ms or longer, comprised three or more spikes and reached an instantaneous frequency of $\geq$ 20 Hz. To examine spike rebound, the frequency of spike activity during the first 415 – 450 ms after termination of the current pulse was monitored. In all recordings, a liquid junction potential of 10 mV was corrected for during analysis.

Histochemistry

Following juxtacellular neurobiotin labelling (see above) fish were removed from the patch clamp setup and transferred to Eppendorf tubes containing 4 % paraformaldehyde (Fisher Scientific) dissolved in phosphate buffered saline (PBS). After a 90 min fixation period, preparations were rinsed with PBSTX (PBS containing 0.1 % Triton X-100) and placed in PBSTX solution containing Cy3-conjugated streptavidin (1:100; Sigma). Fish were incubated in this solution for 12 – 15 h before extensive rinsing in PBSTX and transfer to blocking solution (composition: 3 % milk powder; 1 % DMSO; 0.1 % Triton-X 100 in PBS) containing anti-tyrosine hydroxylase antibody (1:100; Molecular Probes). Fish were incubated in primary antibody for 4 h at room temperature or overnight at 4 °C prior to wash and transferred to fresh blocking solution containing Cy5-labelled secondary antibodies (1:200; Invitrogen). Fish were then rinsed and cleared in glycerol prior to mounting on microscopy slides.

Confocal Microscopy and Laser Ablation

Images were acquired on an Olympus FV1000 confocal laser scanning microscope using Fluoview FV1000 capture software in 1 – 2 µm z-stack increments. For laser ablation studies, 1 dpf (20 – 24 hpf for DC2 ablations; 30 – 32 hpf for DC2/4/5 ablations) *ETvmat2:GFP* transgenic embryos were embedded in 1.5 % low melting point agarose (Melford) dissolved in egg water containing 0.02 % MS-222. Individual neurons were sequentially targeted for ablation by drawing a region of interest around the cell soma using the software features of the confocal microscope. Targeted neurons were irradiated for 20 – 30 s with a UV (405 nm) laser set to 90 % power (50 mW). Successful ablation was confirmed by examination of GFP fluorescence in live fish at around 2 hours post-ablation and with GFP fluorescence/anti-TH immunohistochemistry in fixed fish at 4 dpf, once behavioural recordings had been completed.

Behavioural Experiments

Individual 4 dpf larvae were transferred to a darkened recording chamber that contained an overhead camera (Point Grey DragonFly 2) and an infrared light source for illumination. Fish were allowed to acclimatise for 15 minutes before behaviour was recorded for a period of 10 minutes. Digital video (avi format) recordings were acquired at 15 frames per second using Flycap2 software (Point Grey). Files were then processed in VirtualDub and converted to the micro fly movie format (ufmf) using any2ufmf (`http://ctrax.sourceforge.net/any2ufmf.html`) for subsequent analysis in California Institute of Technology Fly Tracker (Ctrax, v 0.2.16; [S1]) and Janelia Automatic Animal Behavior Annotator (JAABA, v 0.5; [S2]). The position and orientation of individual fish was quantified in a semi-automated manner by Ctrax. Errors in tracking (which occurred at a frequency of $\sim$ 0.23 per fish per minute of video) were subsequently corrected using FixErrors. Using JAABA, the Ctrax-derived position and orientation data were subsequently classified into behavioural categories. JAABA required an initial training period in which examples of a given behaviour and non-behaviour (for example, beat and glide swimming or not beat and glide swimming, respectively) were manually labelled. The machine learning algorithm was then executed and the output (behaviour or non-behaviour) confirmed by manual inspection and retrained if necessary.

Statistical Analysis

Statistical analyses were performed in R v3.1.1. Normality and scedasticity were first tested. To determine statistical significance, the two tailed Student's t-test or Mann Whitney U test was used for normal or non-normal data respectively. Results within the text are presented as mean $\pm$ standard error. Statistical significance is reported as follows: * $p < 0.05$; ** $p < 0.01$; *** $p < 0.001$.

SUPPLEMENTAL REFERENCES

[S1] Branson, K., Robie, A.A., Bender, J., Perona, P., and Dickinson, M.H. (2009). High-throughput ethomics in large groups of Drosophila. Nat. Methods 6, 451-457.

[S2] Kabra, M., Robie, A.A., Rivera-Alba, M., Branson, S., and Branson, K. (2013). JAABA: interactive machine learning for automatic annotation of animal behavior. Nat. Methods 10, 64-7.
